# Knowledge and attitudes about vitamin D, and behaviors related to vitamin D in adults with and without coronary heart disease in Saudi Arabia

**DOI:** 10.1186/s12889-017-4183-1

**Published:** 2017-03-16

**Authors:** Najlaa M. Aljefree, Patricia Lee, Faruk Ahmed

**Affiliations:** 1 Public Health, School of Medicine and Menzies Health Institute Queensland, Griffith University, Building G01, Room 3.30, Gold Coast campus, Gold Coast, QLD 4222 Australia; 2 Department of Medical Research, China Medical University Hospital, China Medical University, Taiwan, Taiwan

**Keywords:** Vitamin D deficiency, Knowledge, Attitudes, Sun exposure, Vitamin D supplements, Saudi Arabia

## Abstract

**Background:**

Vitamin D deficiency is prevailing in Saudi Arabia. Recent national data indicated an inverse association between vitamin D status and coronary heart disease (CHD), which increases concerns about vitamin D deficiency as a serious public health problem. Therefore, the current study aimed to investigate whether knowledge, attitudes and behaviors related to vitamin D contribute to the prevalence of vitamin D deficiency among adults with and without CHD in Saudi Arabia.

**Methods:**

This case-control study consisted of 130 CHD cases and 195 matched controls. The study subjects were recruited from three hospitals in Saudi Arabia. Structured interviews were completed to collect data on participants’ socio-demographics, knowledge about vitamin D, attitudes toward sun exposure, and behaviors related to vitamin D. Also, serum vitamin D levels were measured.

**Results:**

Severe vitamin D deficiency [serum 25(OH)D < 10 ng/mL] was more prevalent in the CHD cases than in the controls (46% and 3%, respectively). The total knowledge score was higher in the controls than in the cases [2.5 (±1.8) and 1.6 (±2.2), respectively]. The cases had better attitudes toward sun exposure compared to the controls (*p* = 0.001); however, the controls had better attitudes toward vitamin D compared to the cases (*p* = 0.001). The controls had a higher consumption of multivitamin supplements than the cases (6.7% and 0.8%, respectively; *p* = 0.010). Similarly, the controls had a higher consumption of butter (*p* = 0.001), oily fish (*p* = 0.004), and liver (*p* = 0.003) than the cases; however, the cases had a significantly higher intake of milk (*p* = 0.001). A multivariate logistic regression showed that vitamin D deficiency [25(OH)D < 20 ng/mL] was associated with low levels of knowledge about vitamin D, with an odds ratio of 1.82 (95% CI: 1.08–3.06, *P* = 0.024). Vitamin D deficiency was also associated with low intake of vitamin supplements, with an odds ratio of 4.35 (95% CI: 2.12–8.92, *P* < 0.001).

**Conclusion:**

The present study revealed that low levels of knowledge about vitamin D and low consumption of vitamin supplementation, including vitamin D, calcium, multivitamin, and calcium supplements with vitamin D, may have contributed to the higher prevalence of vitamin D deficiency among the CHD cases than among the controls. Further studies using a qualitative approach are crucial to explore the underlying reasons for low knowledge about vitamin D and behaviors related to vitamin D including the intake of vitamin supplementation  that may contribute to the high burden of vitamin D deficiency in the country.

**Electronic supplementary material:**

The online version of this article (doi:10.1186/s12889-017-4183-1) contains supplementary material, which is available to authorized users.

## Background

Recent evidence has indicated that vitamin D deficiency and insufficiency are becoming global epidemics [1]. Studies conducted in Western countries have shown that vitamin D deficiency was present in 20% -25% of the total population [2–4]. In the Middle East region, approximately 60%–65% of the population was affected [1]. Vitamin D deficiency also has a significant presence in Saudi Arabia, even though there is plentiful sunlight throughout the year. The majority of studies that have measured vitamin D levels in Saudi Arabia have indicated a high prevalence of vitamin D deficiency among different population groups [5–10]. A recent national survey showed that almost 40% of males and 60% of females in Saudi Arabia had vitamin D deficiency [11].

Aside from the classical role of vitamin D in bone health and the regulation of calcium and bone homeostasis, several large observational studies worldwide have shown an association between vitamin D deficiency and the risk of coronary heart disease (CHD) and associated risk factors such as hypertension and diabetes [12–16]. Furthermore, recent meta-analyses of observational studies also reported significant associations of vitamin D deficiency with cardiovascular disease (CVD) mortality [17], and the increased risk of CVD [18]. Thus, the existing literature of observational studies indicated an association between vitamin D deficiency and the risk of CHD. Nevertheless, to date only a few randomized controlled trials (RCTs) have been conducted to examine the effect of vitamin D supplementation on reducing the risk of CHD [19–21]. However, these studies have failed to demonstrate any causal relationship between vitamin D status and the risk of CHD [19–21]. These studies are flawed with small sample size. Moreover, Mendelian randomization study on the role of vitamin D in CHD illustrated that there is no association between vitamin D deficiency and the risk of CHD [22]. However, this result is only generalizable in European ethnicity but not in Middle Eastern populations. While the casual relationship between vitamin D deficiency and the risk of CHD cannot be determined based on limited number of studies, yet vast literature consistently demonstrated an association between vitamin D deficiency and the risk of CHD.

Exposure to sunlight is the main source of vitamin D, and there are also a few dietary sources of vitamin D, including oily fish and egg yolks, as well as vitamin D dietary supplements [23]. Although the biological factors that reduce serum vitamin D levels are known, the effects of cultural and lifestyle behaviors, as well as knowledge and attitudes about vitamin D, need further investigation. Relatively few studies have assessed knowledge and attitudes in relation to vitamin D worldwide [24–27]. Only one study in Saudi Arabia has examined the knowledge and attitudes about vitamin D [28]; however, the study had limitations such as it was conducted only among college students and with a small sample size and sex restriction (only eight females were involved) [28].

Furthermore, in Saudi Arabia, we have demonstrated the association between vitamin D deficiency [25(OH)D < 20 ng/mL] and the presence of CHD among adults [OR: 6.5, 95% CI: 2.7–15, *p* = < 0.001] [29]. We have also found an association between vitamin D deficiency [25(OH)D < 20 ng/mL] and diabetes among subjects with CHD [OR: 2.9, 95% CI: 1.02–8.5, *p* = 0.04] in Saudi Arabia [30]. Taking into consideration the high rates of CHD and associated risk factors such as obesity, diabetes, hypertension, and hypercholesterolemia in Saudi Arabia [31–34], as well as the high prevalence of vitamin D deficiency in the country [6, 9], there is a need to effectively address these problems. Thus, it is essential to investigate whether knowledge and attitudes regarding vitamin D may play a role in establishing healthy/unhealthy behaviors that contribute to the difference in vitamin D status between CHD patients and subjects without CHD in Saudi Arabia. Therefore, this research aimed to (1) report the prevalence of vitamin D deficiency in subjects with and without CHD, (2) compare the levels of knowledge and attitudes about vitamin D between the two groups, (3) investigate and compare vitamin D-related behaviors in both groups, and (4) to examine the associations of vitamin D status with knowledge, attitudes, and behaviors about vitamin D. This information is expected to provide evidence for developing appropriate health promotions and educational interventions for the general population, thereby increasing knowledge and understanding about the importance of vitamin D and potentially reducing the risk of CHD in Saudi Arabia.

## Methods

### Study population

This case-control study has been described in detail elsewhere [29]. In brief, the current study was conducted in the summertime between May and October 2015 in the cities of Jeddah and Makkah, Saudi Arabia. All included participants were adults of both genders, either Saudis or people who had been residents of Saudi Arabia for at least five years. A total of 152 cases and 236 controls were approached, but 9 cases and 35 controls were ineligible as they did not meet the inclusion criteria. Of the remaining eligible subjects, 13 cases and 6 controls declined to participate in this study. Finally, 130 subjects with CHD (the cases) and 195 subjects without CHD (the controls) were took part in this study. The cases were recruited from the cardiology department at King Abdullah Medical City (KAMC), and the controls were recruited from family medicine clinics and nose and throat clinics at Tunsi private hospital (153 subjects), and ophthalmology clinics at King Abdulaziz University (KAU) hospital (42 subjects). Study participants with medical conditions that may influence vitamin D metabolism, including kidney disease, osteoporosis, liver disease, hyperparathyroidism, and hyperthyroidism, were excluded. All eligible subjects signed written informed consent forms before participating in the study.

Ethical approval was obtained from the Griffith University Human Research Ethics Committee (GU Ref No: MED/59/14/HREC), the Institutional Review Board at KAMC (IRB No: 15–194), and the Research Ethics Committee at KAU (Reference No ll8–15).

### Data collection

All study participants were interviewed in person using a structured questionnaire. Data were collected in relation to participants’ socio-demographic, such as age, gender, marital status, education level, place of residence in Saudi Arabia, nationality, employment, and monthly income. Likewise, data related to behavioral risk factors such as cigarette smoking, water-pipe smoking, and physical activities were also collected during interviews. The definition of a current smoker was a participant who smoked at least one cigarette per day, whereas a previous smoker was defined as a participant who had previously smoked but had quit. A water-pipe smoker was defined as a participant who smoked at least one water-pipe per week at the time of data collection. The practicing exercise was categorized into moderate exercise, such as jogging or walking; vigorous exercise, such as aerobics or bicycling; and sedentary behaviors, such as doing only a little bit of walking outside the home. The structured questionnaire also included three additional sections to collect information on knowledge, and attitudes about, and behaviors toward, vitamin D in Saudi Arabia. Sections one and two gathered data on knowledge and attitudes about vitamin D and sun exposure, and section three gathered data on participants’ behaviors in relation to vitamin D, including sun exposure habits (time spent outdoors during weekdays and weekends, and parts of the body that get exposure to the sun) and use of sun protection. Section three in the questionnaire also asked participants to report the amount and duration of using supplementation, including vitamin D, calcium, multivitamins, and calcium supplements with vitamin D. Questions related to the frequency of intake of some food items rich in vitamin D, such as milk, butter, eggs, oily fish (salmon, tuna, sardines), and liver were also included. Questions related to knowledge and attitudes about, and behaviors toward, vitamin D were adapted from a number of validated questionnaires [24–26] (questions in Additional file 1).

### Biochemical measurements

Blood samples (10 ml) were collected from all study subjects via venipuncture to assess their serum levels of 25(OH)D using chemiluminescence microparticle immunoassay (CMIA) on the Architect system (Abbott). The blood samples were centrifuged at 2000 rpm for 15 min then the serum was separated and was kept frozen at −80 °C while waiting for additional laboratory analyses. All laboratories are certified by the Saudi Ministry of Health and located in the same hospitals where the study took place. The definition of vitamin D deficiency and insufficiency were as serum concentrations of 25(OH)D < 10 ng/mL and 10 to <19.9 ng/mL, respectively, while adequate vitamin D serum level was defined as 25(OH)D ≥ 20 ng/mL [35].

### Statistical analysis

Statistical analyses were accomplished using the Statistical Package for Social Science (SPSS) Version 22. Categorical variables were reported as numbers and percentages. Since there were few subjects in each group who smoked a water-pipe and few subjects were practicing vigorous exercise, cigarette smoking and water-pipe smoking were combined, and moderate exercise was also combined with vigorous exercise as one category. Normality tests were completed for all variables. A chi-square test was used to compare vitamin D status [deficient as serum 25(OH)D < 10 ng/mL, insufficient as serum 25(OH)D 10 to 19.9 ng/mL, and adequate as serum 25(OH)D ≥ 20 ng/mL] between subjects with CHD and subjects without CHD. Likewise, chi-square tests were used to compare knowledge about vitamin D, attitudes toward vitamin D and sun exposure, and vitamin D-related behaviors including sun exposure, the use of sun protection, the use of supplementation, and the intake of food rich in vitamin D between subjects with CHD and subjects without CHD.

The scoring system for knowledge about vitamin D was as follows: study subjects were asked about their knowledge related to vitamin D during the interview by the researcher (NA). Participants who were considered to have a high knowledge level of vitamin D were those who chose the right answers for questions 1, 3, 4, and 5 out of five questions on vitamin D knowledge and were scored according to the total correct answers. Conversely, participants who chose the wrong answers to all of those questions were considered to have a low knowledge level. Similarly, we also calculated the total scores for attitudes (four questions) and behaviors (questions about sun exposure and using of sun protection, the intake of supplements, and the consumption of food rich in vitamin D, respectively) (questions in Additional file 1). After that, we regrouped them using the median of the study sample as a cut-off point to determine the levels of knowledge, attitudes, and three categories of behaviors in order to conduct the multivariate logistic regression.

A Mann-Whitney U test was carried out to compare the difference in total knowledge score between the cases with CHD and the controls. Moreover, a Mann-Whitney U test was also conducted in order to compare the difference in the consumption of food items rich in vitamin D between the two groups, as all food items were not normally distributed. Finally, three multivariate logistic regression models were conducted to examine if there independent associations of vitamin D status with knowledge, attitudes, and vitamin D related behaviors. Because of the small sample size, we combined case and control subjects and controlled for age, gender, and CHD status. In consideration of strong collinearity between CHD and education, employment, citizenship, and marital status identified in our previous studies involving the same sample, these sociodemographic variables were not included in the models. Moreover, because of the small sample size, we combined vitamin D deficiency and insufficiency together to increase the statistical precision; hence, vitamin D deficiency and adequate vitamin D status were defined as [serum 25(OH)D < 20 ng/mL and ≥20 ng/mL, respectively] for the purpose of multivariate logistic regression analysis. A *p* value <0.05 was considered statistically significant.

## Results

The socio-demographic characteristics of study subjects are shown in Table 1. The majority of the cases with CHD and the controls without CHD had similar gender distribution (63% males and 37% females) and were married. In comparison with the controls, a greater proportion of the CHD subjects were 49 years and older (74.6% and 69.7%, respectively), living in rural areas (12.3% and 1%, respectively), and Saudi citizens (81% and 63%, respectively). However, the controls without CHD were more educated, more employed (either full time, part time, or self-employed), and more frequent smokers than the cases with CHD.

**Table 1:** Socio-demographic characteristics and lifestyle behaviors variables among case and control subjects

Variables	Cases (*n* = 130)		Controls (*n* = 195)	
	N	%	N	%
*Age (years)*				
< 49	33	25.4	59	30.3
≥ 49	97	74.6	136	69.7
*Gender*				
Male	82	63	123	63
Female	48	37	72	37
*Marital status*				
Single	7	5.4	34	17.4
Married	91	70	140	71.8
Divorced	32	24.6	21	10.8
*Citizenship*				
Saudis	105	80.8	122	62.6
Non-Saudis	25	19.2	73	37.4
*Place of residence*				
Rural	16	12.3	2	1
Urban	111	85.4	192	98.5
Semi-rural	3	2.3	1	0.5
*Education*				
Up to primary levels	67	51.6	28	14.4
High School & bachelor or diploma degree	61	46.9	156	80
Master or PhD degree	2	1.5	11	5.6
*Employment*				
Employed (Full time, Part time, self-employed)	42	32.3	159	81.5
Unemployed (Student, Retired, House wife)	88	67.7	36	18.5
*Family income (SR* ^a^ */monthly)*				
< 5000	56	43.1	74	37.9
5000–15,000	50	38.5	94	48.2
15,000- ≥ 25,000	24	18.5	27	13.8
*Smoking*				
Current <20 cigarettes/day	23	17.7	39	20
Previous smoker	33	25.4	10	5.1
Non-smoker	74	56.9	146	74.9
*Exercise*				
Never & rarely	44	33.8	84	43.1
1–2 times/week	22	16.9	46	23.6
More than 3–4 times/week	64	49.2	65	33.3

^a^Saudi Riyal (1SR = .37 AUD)

### Prevalence of vitamin D deficiency

**Fig. 1:**
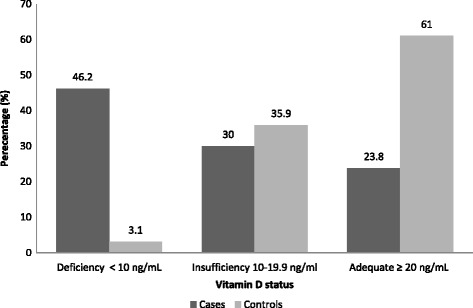
Vitamin D status among case and control subjects

Figure 1 shows vitamin D status in subjects with and without CHD. There was a significant difference between the two groups with respect to vitamin D status (*p* = < 0.001). Over 46% of the CHD cases were classified as having a vitamin D deficiency [serum 25(OH)D < 10 ng/mL], whereas only 3% of the controls had a vitamin D deficiency. Likewise, the majority of the control subjects had adequate vitamin D levels [serum 25(OH)D ≥ 20 ng/mL] in contrast to the CHD cases (61% and 24%, respectively).

### Knowledge about vitamin D

Table 2 illustrates knowledge about vitamin D between the cases with CHD and the controls without CHD. There was a significant difference between the groups related to knowledge about vitamin D. Almost 70% of the controls have heard or learned about vitamin D compared to only 40% of the CHD cases (*p* = 0.001). Doctors and friends/relatives were the main source of information about vitamin D in both groups. Also, half of the controls knew that vitamin D is important for bone health, compared to 31% of the cases (*p* = 0.003). Similarly, half of the controls knew that exposure to sunlight is the main source of vitamin D, compared to only 29% of the cases (*p* = 0.001). In addition, a quarter of the controls admitted that oily fish is a good food source for vitamin D, compared to only 10% of the cases (*p* = 0.001). Similarly, 10% of the controls and 4% of the cases admitted that milk is a good food source for vitamin D. The total knowledge score was higher in the controls than in the cases [2.5 (±1.8) and 1.6 (±2.2), respectively].

**Table 2:** knowledge regarding vitamin D stratified by case and control groups

Variables	Cases (*n* = 130)		Controls (*n* = 195)		*P*-value*
	N	%	N	%	
Have you ever heard/learnt about vitamin D?					
Yes	53	40.8	136	69.7	.001
No	77	59.2	59	30.3	
Where have you heard or learnt about vitamin D?					
Newspaper/Magazine	1	0.8	4	2.1	.001
TV	2	1.5	8	4.1	
Doctor	28	21.5	42	21.5	
Friends/Relatives	16	12.3	45	23.1	
School/university	3	2.3	22	11.3	
Internet	3	2.3	7	3.6	
Other health professionals (dietician)	0	0	10	5.1	
I don’t know	77	59.2	57	29.2	
Vitamin D helps which of the following health effects?					
Prevention of kidney disease	0	0	2	1	.003
Healthy bones	41	31.5	99	50.8	
Prevention of cancer	3	2.3	2	1	
I don’t know	86	66.2	92	47.2	
Where do you think the body gets vitamin D from?					
Diet	7	5.4	18	9.2	.001
Sun exposure	38	29.2	98	50.8	
Supplements	3	2.3	5	2.6	
I don’t know	82	63.1	74	37.9	
What type of food is a good source of vitamin D?					
Vegetables & fruits	3	2.3	28	14.4	.001
Milk	5	3.8	19	9.7	
Fatty fish (salmon, sardines)	14	10.8	49	25.1	
Olive oil	0	0	3	1.5	
Eggs	8	6.2	14	7.2	
I don’t know	100	76.9	82	42.1	
Knowledge total score^a^	1.6 (± 2.2)		2.5 (± 1.8)		.001

*P-value based on X^2^ -test

^a^Numbers refer to mean and standard deviation for each group, *P*-value based on Mann-Whitney U test

### Attitudes toward vitamin D

Table 3 illustrates attitudes toward vitamin D stratified by case and control groups. The controls had better attitudes about vitamin D as more than 80% of the controls responded yes to the question of whether vitamin D was important to health, in compared to only 46% of the cases (*p* = 0.001). However, the cases with CHD had better attitudes toward sun exposure as 48% of the cases reported that they like to be exposed to sunlight all the time, compared with only 18% of the controls. At the same time, 18% of the controls stated that they avoid sunlight, compared with 11% of the cases (*p* = 0.001). In addition, more cases than controls agreed with the statement, “I’m concerned that my current vitamin D level might be too low” (65% and 51%, respectively) (*p* = 0.001). All of the cases and the majority of the controls (92%) did not use a parasol to shade themselves from sunlight (*p* = 0.001).

**Table 3:** Attitudes toward vitamin D stratified by case and control groups

Variables	Cases (*n* = 130)		Controls (*n* = 195)		*P*-value*
	N	%	N	%	
Do you think vitamin D is important for your health?					
Yes	61	46.9	159	81.5	**.001**
No	2	1.5	17	8.7	
I don’t know	67	51.5	19	9.7	
How do you feel about sun exposure?					
I like to expose to sunlight all the time	62	47.7	34	17.4	**.001**
I like to expose to sunlight sometimes	47	36.2	101	51.8	
I rarely expose to sunlight	6	4.6	26	13.3	
I avoid expose to sunlight	15	11.5	34	17.4	
Do you often use a parasol to shade from the sun?					
Yes	0	0	15	7.7	**.001**
No	130	100	180	92.3	
How much do you agree or disagree with the following statement: “I’m concerned that my current vitamin D level might be too low”					
Disagree	10	7.7	41	21	**.003**
Neither agree or disagree	35	26.9	53	27.2	
Agree	85	65.4	101	51.8	

*P-value based on X^2^ -test

### Vitamin D-related behaviors

Table 4 illustrates vitamin D-related behaviors stratified by case and control groups. Regarding sun exposure behaviors, the majority of subjects in the case and control groups worked indoors (81% and 97%, respectively) (*p* = 0.001). A high proportion of controls were exposed to sunlight for less than 30 min per day (64.6% and 44.6%, respectively) (*p* = 0.001). Also, more cases than controls had sufficient sun exposure (30–60 min or more per day) during weekdays (37.7% and 25.2%, respectively) (*p* = 0.001). Likewise, more cases than controls were sufficiently exposed to sunlight (less than 30 min and 30–60 min per day or more) during weekends (64.6% and 51.3%, respectively) (*p* = 0.001). However, 49% of the controls and 35% of the cases do not spend time outdoors exposed to sunlight at all during weekends (*p* = 0.001). The majority of cases and controls only exposed their faces and hands to sunlight (73% and 80%, respectively); however, a larger proportion of the cases exposed both arms to the sunlight compare to the controls (18% and 5%, respectively) (*p* = 0.001). In addition, 20% of the controls reported using sun protection 1–4 times per week and more than five times per week, compared with only 0.8% of the cases (*p* = 0.001). The results also showed that more controls than cases were consuming multivitamin supplements (6.7% and 0.8%, respectively) (*p* = 0.010). There were no significant differences between the case and control groups regarding the consumption of vitamin D supplements, calcium supplements, and calcium supplements with vitamin D (all *p* values >0.05).

**Table 4:** Vitamin D related behaviors stratified by case and control groups

Variables	Cases (*n* = 130)		Controls (*n* = 195)		*P*-value*
	N	%	N	%	
Sun exposure and using of sun protection					
Do you work mainly:					
Indoor	106	81.5	191	97.9	**.001**
Outdoor	24	18.5	4	2.1	
How much time do you often spend outdoors per day on weekdays?					
Not at all	23	17.7	20	10.3	**.001**
˂30 min	58	44.6	126	64.6	
30–60 min	27	20.8	45	23.1	
> 60 min	22	16.9	4	2.1	
How much time do you often spend outdoors per days on weekends?					
Not at all	46	35.4	95	48.7	**.001**
˂30 min	48	36.9	46	23.6	
30–60 min	19	14.6	47	24.1	
> 60 min	17	13.1	7	3.6	
Which parts of your body get exposed to the sun?					
Face	0	0	0	0	**.001**
Hand	12	9.2	29	14.9	
Face & hand	95	73.1	156	80	
Both arms	23	17.7	10	5.1	
Both legs	0	0	0	0	
Completely covered	0	0	0	0	
How often do you wear sunscreen while outdoors in the sun?					
Never	129	99.2	156	80	**.001**
1–4 times/week	0	0	23	11.8	
> 5 times/week	1	0.8	16	8.2	
The use of supplementation					
Do you take vitamin D supplements					
Yes	18	13.8	21	10.8	.403
No	122	86.2	174	89.2	
Do you take calcium supplements?					
Yes	4	3.1	9	4.6	.488
No	126	96.9	186	95.4	
Do you take multivitamin supplements?					
Yes	1	0.8	13	6.7	**.010**
No	129	99.2	182	93.3	
Do you take calcium supplements with vitamin D?					
Yes	1	0.8	5	2.6	.239
No	129	99.2	190	97.4	
The intake of food rich in vitamin D					
How often do you drink milk?					
Never	33	25.4	82	42.1	**.002**
1–2 times/week	27	20.8	43	22.1	
3–6 times/week	27	20.8	37	19	
≥ once/day	43	33.1	33	16.9	
How often do you eat butter?					
Never	113	86.9	132	67.7	**.001**
1–2 times/week	12	9.2	40	20.5	
3–6 times/week	4	3.1	20	10.3	
≥ once/day	1	0.8	3	1.5	
How often do you eat eggs?					
Never	21	16.2	32	16.4	.998
1–2 times/week	95	73.1	142	72.8	
3–6 times/week	14	10.8	21	10.8	
≥ once/day	0	0	0	0	
How often do you eat oily fish (salmon, tuna, sardine)?					
Never	66	50.8	72	36.9	**.034**
1–2 times/week	44	33.8	73	37.4	
3–6 times/week	14	10.8	41	21	
≥ once/day	6	4.6	9	4.6	
How often do you eat liver?					
Never	106	81.5	125	64.1	**.001**
1–2 times/week	15	11.5	52	26.7	
3–6 times/week	7	5.4	18	9.2	
≥ once/day	2	1.5	0	0	

*P-value based on X^2^ -test

Moreover, the consumption of food items that are rich in vitamin D was also compared between the two study groups. Table 5 presents the difference in the intake of some food items that are rich in vitamin D between the cases and controls. Drinking milk was more common among the cases than the controls as 54% of the cases were drinking milk regularly (3–6 times a week, or once a day or more), compared with only 36% of the controls (*p* = 0.002). However, eating butter (*p* = 0.001), oily fish (*p* = 0.034), and liver (*p* = 0.001) was more common among the controls than the cases. Approximately 12% of the controls had a high consumption of butter, compared with only 3.9% of the cases (*p* = 0.001). In addition, 25.6% of the controls had a high consumption of oily fish, compared with 15.4% of the cases (*p* = 0.034). Furthermore, 9.2% of the controls had a high consumption of liver, compared with 6.9% of the cases (*p* = 0.001). There was no significant difference in the consumption of eggs between the two groups (*p* > 0.05). Similarly, the cases had a significantly higher intake of milk (*p* = 0.001) than the controls. Conversely, the controls had a higher consumption of butter (*p* = 0.001), oily fish (*p* = 0.004), and liver (*p* = 0.003).

**Table 5:** Differences in intake of food items that rich in vitamin D between cases and controls

Food items	Cases		Controls		Mann-Whitney U	Z-value	*P*-value*
	Median	Range	Median	Range			
Milk	0.50	0–3	0.28	0–3	9700.5	-3.701	**.001**
Butter	0	0–1	0	0–2	10157.0	-4.018	**.001**
Eggs	0.28	0–1	0.28	0–1	12205.5	-0.581	.561
Oily fish	0	0–1	0.14	0–2	10399.0	-2.877	**.004**
Liver	0	0–1	0	0–0.79	10700.5	-2.978	**.003**

* P-value based on Mann-Whitney U test

### Associations of vitamin D status with knowledge, attitudes, and behaviors about vitamin D

We conducted the multivariate logistic regression analysis to examine the independent associations of vitamin D status with knowledge, attitudes, and behaviors about vitamin D in the study subjects after controlling for potential confounders including CHD. Table 6 illustrates the results of the multivariate logistic regression modelling (Model 1 - Model 3). Low levels of knowledge about vitamin D was significantly associated with vitamin D deficiency [25(OH)D < 20 ng/mL] *(P* = 0.024). After adjustment for age, gender, and CHD, subjects with lower levels of knowledge about vitamin D were 1.82 times more likely to suffer from vitamin D deficiency compared to those with higher levels of knowledge about vitamin D (OR: 1.82, 95% CI: 1.08–3.06). Furthermore, low intake of vitamin supplements, including vitamin D supplements, calcium supplements, multivitamin supplements, and calcium supplements with vitamin D, was significantly associated with vitamin D deficiency *(P* < 0.000). After adjustment for age, gender, and CHD, subjects with lower intake of vitamin supplements were 4.35 times more likely to suffer from vitamin D deficiency compared to those with higher intake of vitamin supplements (OR: 4.35, 95% CI: 2.12–8.92). No significant associations were detected between vitamin D deficiency and attitudes about vitamin D, behaviors regarding sun exposure and using sun protection, and the consumption of food rich in vitamin D.

**Table 6:** Results of Multivariate Logistic Regression Analysis

	Adjusted OR^a^ (95% CI)	*P*-value
Model 1: Knowledge about vitamin D		
High knowledge levels	1.00 (referent)	
Low knowledge levels	1.82 (1.08–3.06)	**0.024**
Model 2: Attitudes toward vitamin D		
Good attitude	1.00 (referent)	
unfavorable attitude	0.96 (0.58–1.59)	0.899
Model 3: Vitamin D related behaviors		
*Sun exposure and using of sun protection*		
High score	1.00 (referent)	
Low score	1.54 (0.87–2.71)	0.132
*Intake of vitamin supplements*		
High intake	1.00 (referent)	
Low intake	4.35 (2.12–8.92)	**<.000**
*Consumption of food rich in vitamin D*		
High intake	1.00 (referent)	
Low intake	0.87 (0.53–1.4)	0.612

^a^Multivariate Logistic Regression model after adjustment for age, gender, and CHD

## Discussion

The current study revealed a number of important findings. First, the cases with CHD had a higher prevalence of vitamin D deficiency compared with the controls. Second, knowledge of various aspects of vitamin D was lower among the CHD cases than the controls. Third, the cases with CHD had a better attitudes toward sun exposure compared with the controls; however, the controls had better attitudes toward vitamin D compared to the cases. Fourth, a higher proportion of the CHD cases were sufficiently exposed to sunlight during weekdays and weekends. Almost three-quarters of the subjects in both groups were only exposing their faces and hands to sunlight. Fifth, the controls had a higher intake of multivitamin supplements and a higher consumption of butter, oily fish, and liver compared with the CHD cases, while milk intake was higher among the CHD cases than the controls. Finally, after controlling for potential confounding factors, low levels of knowledge about vitamin D and the low intake of vitamin supplements were significantly associated with vitamin D deficiency.

The study findings demonstrated that vitamin D deficiency was significantly more prevalent in the CHD cases than the controls. Previous studies have reported similar results [36, 37]. Based on these findings, the present study attempted to answer an important question, which are whether the higher prevalence of vitamin D deficiency in the CHD cases compared with the controls is due to differences in knowledge, attitudes, and vitamin D-related behaviors in both groups?. To the best of our knowledge, this is the first study that has compared the knowledge and attitudes about, and behaviors toward, vitamin D between subjects with and without CHD. It is also the first study to examine the associations between vitamin D status and knowledge, attitudes, and behaviors about vitamin D in Saudi Arabia.

The traditional knowledge, attitudes, and practice (KAP) survey theory suggests a direct linear relationship between knowledge, attitudes, and behaviors, which is, according to several studies, very simple and not true [38]. This is because people’s behaviors have a multifactorial nature and depend on many factors such as socio-cultural and environmental factors, not just knowledge and attitudes [38]. Thus, our study showed inconsistent findings between knowledge, attitudes, and behaviors in both groups.

The present study showed that the controls had higher levels of knowledge about vitamin D compared with the CHD cases. The total score of knowledge about vitamin D was higher in the controls than in the cases, including understanding the importance of vitamin D in disease prevention and knowledge of sources of vitamin D, such as sun exposure and certain foods. This difference in knowledge between the cases and controls may be due to the fact that the control subjects were more educated than the cases. These results are consistent with the multivariate logistic regression results that showed a significant association between low levels of knowledge about vitamin D and vitamin D deficiency in our sample after controlling for CHD. A study among older adults in Netherlands has reported similar results as the higher levels of knowledge about vitamin D was associated with higher vitamin D serum levels [27].

Overall, the present study showed a lack of knowledge about vitamin D in both groups, but more specifically in the CHD cases. Approximately one-third of the controls and two-thirds of the cases have never heard or learned about vitamin D. In addition, of those who reported that they have heard about vitamin D, 38% of the controls and 63% of the cases reported that they did not know any of the vitamin D sources, including the role of sun exposure in production of vitamin D. Moreover, there was a confusion about dietary sources of vitamin D among those who reported diet as a source of vitamin D as only a few subjects knew some of the richest sources of dietary vitamin D, such as milk (4% of the cases and 10% of the controls) and fatty fish (11% of the cases and 25% of the controls). Evidence to date has also indicated low levels of knowledge about vitamin D among different populations. A study conducted in the UK showed that approximately one-third of the study participants had never heard about vitamin D, especially older participants [24]. Likewise, low levels of knowledge about vitamin D have been reported in Chinese women [25]. Similarly, a survey in the Netherlands revealed that only 38% of survey participants had heard about vitamin D [27]. Relatively better knowledge about vitamin D has been reported in Australia. A survey conducted in Queensland showed that 69% of the participants knew about vitamin D, and almost 50% of them knew its role in protecting bone health [26]. In Kuwait, a Gulf country, a cross-sectional survey indicated low levels of knowledge about vitamin D among the Kuwaiti population [39].

With respect to attitudes toward vitamin D and sun exposure, almost half of the cases responded “I do not know” to whether vitamin D was important for health, compared to 80% of the controls responding “yes” to the importance of vitamin D for general health. This might be partly due to the higher level of knowledge among the control subjects. However, results showed that the CHD cases had better attitudes toward sun exposure than the controls as a large majority of the CHD cases said, “I like to expose all the time and/or sometimes to sunlight”, whereas a higher proportion of the controls said “I avoid exposure to or rarely expose myself to sunlight”. Similarly, only half of the controls and 65% of the cases were concerned about their current vitamin D status. These results indicated three important points. First, the controls had better attitudes toward vitamin D than the cases. Second, the cases had better attitudes toward sun exposure than the controls, even though they were less knowledgeable about vitamin D. Third, in general, our study sample had an unfavorable attitude toward vitamin D and sun exposure, with a lack of awareness about the importance of vitamin D and exposure to sunlight. Negative attitudes toward sun exposure have been reported among Arabic Gulf populations [39]. Previous studies have also reported negative attitudes toward sun exposure, even among subjects who were considered knowledgeable about vitamin D [25]. This is similar to our findings, as the current study highlighted contradictory results between knowledge about vitamin D and attitudes toward sun exposure. The control subjects had higher levels of knowledge about sun exposure as the main source of vitamin D; however, one-third of the controls had negative attitudes toward sun exposure and stated that they avoided or rarely exposed themselves to sunlight, which may suggest that being knowledgeable about vitamin D does not necessary influence attitudes toward exposure to sunlight as the major source of vitamin D. Furthermore, one possible explanation for cases having better attitudes toward sun exposure than the controls might be due to the interrelationship between attitudes and other variables, such as beliefs [38]. This means cases might answer what they think it is correct or healthy, as the majority of patients are trying to act healthier after being affected by a disease.

Regarding vitamin D-related behaviors, findings related to exposure to sunlight in our study showed that even though a higher percentage of the CHD cases were sufficiently exposed to sunlight, a large percentage of the subjects in each group were not exposed to sunlight during weekdays (17.7% of the cases and 10.3% of the controls) and weekends (35.4% of the cases and 48.7% of the controls). Additionally, more than three-quarters of the participants in both groups only exposed their faces and hands to sunlight, which indicates that very small parts of their bodies were exposed to sunlight for a limited time during the day; hence, our results showed poor sun exposure behaviors among the study subjects, which explain why we did not find a significant association between vitamin D status, and sun exposure behavior in our study. Moreover, the reason the controls had lower levels of exposure to sunlight during weekdays might be due to the higher rate of employment among the controls compared to the CHD cases, which means the controls had longer hours of working at indoor offices and thus, less sun exposure during weekdays.

The current results also indicated limited consumption of vitamin D supplements and multivitamin supplements by the study subjects in general. Higher consumption of vitamin D supplements has been reported in different populations [40]. The use of vitamin D supplements has a significant effect on vitamin D serum levels, especially among those who were rarely exposed to sunlight. The study results showed that the controls had a higher consumption of multivitamin supplements than the cases, which might have affected their vitamin D status. This result is consistent with the results of the multivariate logistic regression as it reported a significant association between vitamin D deficiency and the low intakes of vitamin supplements, including vitamin D supplements, calcium supplements, multivitamin supplements, and calcium supplements with vitamin D.

The consumption of foods rich in vitamin D including butter, oily fish, and liver was significantly higher in the controls than in the cases, except for milk. Overall, consumption of milk was relatively low in our sample, as 42% of the controls and a quarter of the cases reported never drinking milk on a weekly basis. The Ministry of Health in Saudi Arabia fortified fresh milk, powdered milk, and buttermilk with vitamin D in order to reduce the high burden of vitamin D deficiency [41]. Previous studies have also reported low milk consumption in the Saudi population [42]. Furthermore, the consumption of oily fish was low in our sample, especially among the CHD cases, even though Jeddah and Makkah are located on the coast. The poor consumption of butter, fish, and liver among the cases might be due to changes in their dietary patterns after being affected by CHD. Furthermore, results of the regression analysis did not show a significant association between vitamin D deficiency and the low consumption of food rich in vitamin D in our study sample, which may be due to the fact that only 10–20% of vitamin D in human bodies is obtained from food sources [23].

The current study has several limitations. First, the study sample was small. However, the cases and controls were selected from three different hospitals in the two main cities in the western region of the kingdom; hence, it is expected that the results of the study are likely to be generalizable to Saudis living in the western region. Second, the current study did not investigate the reasons for avoiding sunlight and for the poor consumption of vitamin D supplements and/or foods rich in vitamin D among study subjects. Moreover, courtesy bias might be a weakness of this survey as participants may want to give answers that they believe the researcher want to hear. For example, a large number of cases (65%) answered they are concerned about their vitamin D status, even though about two-third of them never heard or learnt about vitamin D. On the other hand, there are limited studies of knowledge and attitudes about, and behaviors toward, vitamin D in Saudi Arabia and the Middle East region. The strength of this study was that no previous studies have compared knowledge and attitudes about, and behaviors toward vitamin D between subjects with and without CHD as well as examined the associations between vitamin D status and knowledge, attitudes, and behaviors about vitamin D in Saudi Arabia.

## Conclusions

In conclusion, the present study showed that vitamin D deficiency was highly prevalent in subjects with CHD than in the controls. Knowledge about vitamin D was higher among the controls, and they had a higher intake of multivitamin supplements and a higher consumption of butter, oily fish, and liver, while the CHD cases had a higher intake of milk and were sufficiently exposed to sunlight during weekdays and weekends. Our findings, thus, suggest that low levels of knowledge about vitamin D and the low consumption of vitamin supplementations, including vitamin D, calcium, multivitamin, and calcium supplements with vitamin D, may have contributed to the high prevalence of vitamin D deficiency among the CHD cases. Although knowledge, attitudes, and behaviors may not be strongly associated with each other in this study, the results have provided valuable information for prevention of vitamin D deficiency, which may contribute to future interventions of CHD. Moreover, additional studies using qualitative approaches are essential to explore the underlying reasons for low knowledge about vitamin D and behaviors related to vitamin D including vitamin D supplementation that might have contributed to the high burden of vitamin D deficiency in Saudi Arabia.

## Additional file

Additional file 1: Questions regarding knowledge and attitudes about vitamin D, and behaviors related to vitamin D in Saudi Arabia. (DOCX 20 kb)

## Abbreviations

25(OH)D: 25-hydroxyvitamin D
CHD: Coronary artery disease
CVD: Cardiovascular disease
KAMC: King Abdullah Medical City
KAP: Knowledge, attitudes, and practice
KAU: King Abdul Aziz University Hospital
RCTs: Randomized controlled trials
