# Supplementary material for: Knowledge and attitudes about vitamin D, and behaviors related to vitamin D in adults with and without coronary heart disease in Saudi Arabia
Source: BMC Public Health. 2017 Mar 16;17:266. doi: 10.1186/s12889-017-4183-1 (PMC5356251; doi:10.1186/s12889-017-4183-1)
Supplement: Additional file 1: — Questions regarding knowledge and attitudes about vitamin D, and behaviors related to vitamin D in Saudi Arabia. (DOCX 20 kb) [file 12889_2017_4183_MOESM1_ESM.docx]

**Additional file 1**

Knowledge about vitamin D:

1. Have you ever heard/learnt about vitamin D?

□Yes □No

1. Where have you heard or learnt about vitamin D?

□ Newspaper/Magazine □ Radio □ TV □ Doctor

□ Friends/Relatives □ School/university □ Internet

□ Other health professionals (dietician) □ I don’t know

1. Vitamin D helps which of the following health effects? (tick all that apply)

□ Prevention of kidney disease □ Healthy bones

□ Prevention of cancer □ I don’t know

1. Where do you think the body gets vitamin D from? (tick all that apply)

□Diet □Sun exposure □supplements □ I don’t know

1. What type of food is a good source of vitamin D? (tick all that apply)

□ Vegetables & Fruits □ Milk □ Fatty fish (salmon, sardines)

□ Olive oil □ Eggs □ I don’t know

Attitudes toward vitamin D:

1. Do you think vitamin D is important for your health?

□Yes □No □ I don’t know

1. How do you feel about sun exposure?

□ I like to expose to sun light all the time

□ I like to expose to sun light sometimes

□ I rarely expose to sun light

□I avoid expose to sun light

1. Do you often use a parasol to shade from the sun?

□Yes □No

1. How much do you agree or disagree with the following statement: “I’m concerned that my current vitamin D level might be too low”.

□ Disagree

□ Neither agree or disagree

□ Agree

Vitamin D related behaviours:

1. Do you work mainly:

□ Indoors □ Outdoors

1. How much time do you often spend outdoors per day on weekdays?

□ Not at all □ ˂30 min □30-60 min

□60-90 min □>90 min

1. How much time do you often spend outdoors per days on weekends?

□ Not at all □ ˂30 min □30-60 min

□60-90 min □>90 min

1. Which parts of your body get exposed to the sun? (tick all that apply)

□ Face □ Hand □ Face & hand

□ both arms □ both legs □ completely covered

1. How often do you wear sunscreen while outdoors in the sun?

□ Never □ 1-2 times/week □ 3-4 times/week

□ 5-6 times/week □ always

1. Do you take vitamin D supplements?

□Yes □No

1. Do you take calcium supplements?

□Yes □No

1. Do you take multivitamin supplements?

□Yes □No

1. Do you take calcium supplements with vitamin D?

□Yes □No

1. How often do you drink milk?

□never □once/week □2 times/week □3-4 times/week

□5-6 times/week □once/day □2 times/day □≥3 times/day

1. How often do you eat butter?

□never □once/week □2 times/week □3-4 times/week

□5-6 times/week □once/day □2 times/day □≥3 times/day

1. How often do you eat eggs?

□never □once/week □2 times/week □3-4 times/week

□5-6 times/week □once/day □2 times/day □≥3 times/day

1. How often do you eat oily fish (salmon, tuna, sardine)?

□never □once/week □2 times/week □3-4 times/week

□5-6 times/week □once/day □2 times/day □≥3 times/day

1. How often do you eat liver?

□never □once/week □2 times/week □3-4 times/week

□5-6 times/week □once/day □2 times/day □≥3 times/day
